# Metabolic Reprogramming in Mutant IDH1 Glioma Cells

**DOI:** 10.1371/journal.pone.0118781

**Published:** 2015-02-23

**Authors:** Jose L. Izquierdo-Garcia, Pavithra Viswanath, Pia Eriksson, Myriam M. Chaumeil, Russell O. Pieper, Joanna J. Phillips, Sabrina M. Ronen

**Affiliations:** 1 Department of Radiology and Biomedical Imaging, University of California San Francisco, San Francisco, California, United States of America; 2 Department of Neurological Surgery, Helen Diller Research Center, University of California San Francisco, San Francisco, California, United States of America; 3 Brain Tumor Research Center, University of California San Francisco, San Francisco, California, United States of America; Swedish Medical Center, UNITED STATES

## Abstract

**Background:**

Mutations in isocitrate dehydrogenase (IDH) 1 have been reported in over 70% of low-grade gliomas and secondary glioblastomas. IDH1 is the enzyme that catalyzes the oxidative decarboxylation of isocitrate to α-ketoglutarate while mutant IDH1 catalyzes the conversion of α-ketoglutarate into 2-hydroxyglutarate. These mutations are associated with the accumulation of 2-hydroxyglutarate within the tumor and are believed to be one of the earliest events in the development of low-grade gliomas. The goal of this work was to determine whether the IDH1 mutation leads to additional magnetic resonance spectroscopy (MRS)–detectable changes in the cellular metabolome.

**Methods:**

Two genetically engineered cell models were investigated, a U87-based model and an E6/E7/hTERT immortalized normal human astrocyte (NHA)-based model. For both models, wild-type IDH1 cells were generated by transduction with a lentiviral vector coding for the wild-type IDH1 gene while mutant IDH1 cells were generated by transduction with a lentiviral vector coding for the R132H IDH1 mutant gene. Metabolites were extracted from the cells using the dual-phase extraction method and analyzed by ^1^H-MRS. Principal Component Analysis was used to analyze the MRS data.

**Results:**

Principal Component Analysis clearly discriminated between wild-type and mutant IDH1 cells. Analysis of the loading plots revealed significant metabolic changes associated with the IDH1 mutation. Specifically, a significant drop in the concentration of glutamate, lactate and phosphocholine as well as the expected elevation in 2-hydroxyglutarate were observed in mutant IDH1 cells when compared to their wild-type counterparts.

**Conclusion:**

The IDH1 mutation leads to several, potentially translatable MRS-detectable metabolic changes beyond the production of 2-hydroxyglutarate.

## Introduction

The unfavorable biological characteristics of brain tumors mean that they correspond to the third highest cancer-related death in patients under the age of 35. The World Health Organization classifies brain tumors histologically according to their dominant cell type and grade. Grade I gliomas are benign. Grade II astrocytomas or oligodendrogliomas are considered low grade, but they are poorly circumscribed, infiltrate into adjacent normal brain, and in most cases progress to a higher-grade glioma. Grade III anaplastic gliomas and Grade IV glioblastomas (GBMs) are classified as high-grade tumors. A new diagnostic paradigm emerged in 2009 when mutations in the cytosolic isocitrate dehydrogenase 1 (IDH1) enzyme were detected in 70–80% of grade II, III and grade IV secondary (upgraded) GBMs, but rarely in primary GBM tumors [1]. Mutations in the mitochondrial isoform of IDH, namely IDH2, have also been reported but these occur more commonly in acute myeloid leukemia (AML) [2]. In addition to glioma and AML, IDH mutations have been confirmed in enchondroma [3] and in some cases of thyroid carcinomas, cartilaginous tumors and intrahepatic cholangiocarcinomas [4,5].

Different studies have analyzed the role of IDH mutations in cancer [5,6]. The main biochemical alteration associated with these mutations is the gain of a new enzymatic activity wherein mutant IDH reduces α-ketoglutarate (α-KG, also called 2-oxoglutarate) to 2-hydroxyglutarate (2-HG). This is in contrast to wild-type IDH, which catalyzes the NADP^+^-dependent oxidative decarboxylation of isocitrate into α-KG. As a result, mutant IDH leads to elevated levels of 2-HG in tumor cells [7]. The exact mechanism through which mutant IDH and 2-HG induce oncogenesis continues to be investigated. Nonetheless, it has been shown that 2-HG acts as a competitive inhibitor of several α-KG-dependent dioxygenases leading to inhibition of histone demethylases and TET family 5-methylcytosine hydroxylases. This leads to genome-wide alterations in histone and DNA methylation and likely mediates tumor development [8–10].

A previous publication using mass spectrometric studies of cellular models suggests that the IDH1 mutation might also cause changes in global cellular metabolism [11]. ^1^H magnetic resonance spectroscopy (MRS) is an alternate and clinically translatable approach for probing metabolite levels. ^1^H MRS has been applied to investigations of glioma patient biopsies and has confirmed that, in addition to elevated levels of 2-HG, other metabolic alterations occur in mutant IDH tumors [12]. Studies of genetically-defined cell models combined with MRS-based metabolomics can be used to further investigate the broad-based metabolic alterations specifically associated with the IDH mutation and its distinct molecular phenotype [13]. Such untargeted metabolic profiling approaches coupled with robust chemometric analysis based on multivariate statistical analysis have been previously employed in cell model systems to investigate the metabolic effects of drug treatments or different genetic phenotypes [14–16]. The complexity and size of the metabolic data require the application of appropriate multivariate statistical methods to identify the most prominent changes in the metabolic signature. Principal component analysis (PCA) is one of the most common analytical techniques in multivariate analysis. Importantly, PCA is a completely unsupervised method that does not require any *a priori* information about the data, allowing for a completely unbiased analysis of the datasets [17,18].

The aim of our study was to analyze the metabolic changes associated with the IDH1 mutation using a comprehensive and reproducible metabolomics platform. To achieve this objective, we analyzed two genetically engineered IDH1 mutant cell models, a U87 glioblastoma cell line-based model and an E6/E7/hTERT immortalized Normal Human Astrocyte (NHA)-based model. MRS data were analyzed by multivariate analysis techniques in order to identify the metabolic differences between wild-type and mutant IDH1 cells common to both cellular models.

## Material and Methods

### Cell culture

U87 glioblastoma cells were obtained from the American Type Culture Collection via the University of California San Francisco Cell Culture Facility (San Francisco, CA, USA). NHAs were generated in the Pieper laboratory [19]. For both models, wild-type IDH1 cells (IDHwt) were generated by transduction with a lentiviral vector coding for the wild-type IDH1 gene and mutant IDH1 cells (IDHmut) were generated by transduction with a lentiviral vector coding for the mutant IDH1 gene (point mutation in R132H) [20]. All our experiments were conducted at a passage level ≥ 15. Untransfected U87 and NHA parental cell lines were also studied as controls.

Cell cultures were maintained in exponential growth in high-glucose Dulbecco’s modified Eagle’s medium (DMEM; UCSF Cell Culture Facility) supplemented with 10% heat-inactivated fetal bovine serum, 2 mM L-glutamine, 100 units/mL penicillin, 100 mg/mL streptomycin and cultured in a humidified atmosphere of 5% CO_2_ in air at 37°C.

### Western blot analysis

Approximately 1x10^7^ cells (cell number determined by visual counting in hemocytometer) were lysed using cell lysis buffer (Cell Signaling, MA, USA) containing protease inhibitors (Millipore, MA, USA). Protein concentration was estimated using the Bradford method in order to load equivalent amounts of protein for western blotting. Proteins were resolved using polyacrylamide gel electrophoresis (BioRad) under denaturing conditions, followed by transfer to PVDF membranes (Millipore, MA, USA). Membranes were blocked overnight in 5% milk (Santa Cruz Biotechnology, TX, USA) in TBST (Tris-buffered Saline with 0.1% Tween 20, pH 7.5) at 4°C. Membranes were then washed 3 times for 5 min each in TBST and incubated with primary antibodies diluted in TBST [IDH1 wild-type (Cell Signaling, MA, USA), IDH1 R132H mutant (Dianova, Hamburg, Germany), β- tubulin (Cell Signaling, MA, USA)] for 1 h at room temperature. Following 3 washes of 10 min each with TBST, HRP-conjugated secondary antibodies (IDH1 mutant (Abcam, MA, USA), IDH1 wild-type and β- tubulin (Cell Signaling, MA, USA) were added for 1 h in TBST at room temperature. Membranes were washed thrice in TBST as described above and developed onto autoradiographic film using an enhanced chemiluminescence substrate kit (Thermo Scientific, MA, USA).

### Growth rate and Cell cycle analysis

For measurement of growth rate, cells were seeded at an initial concentration of 1–2 x 10^6^ cells per flask. Following trypsinization, cells were counted at 4 different time points approximately 24 h apart and doubling time calculated (http://www.doubling-time.com/compute.php). Cell cycle profiles were analyzed by propidium iodide staining and flow cytometry. Approximately 1x10^6^ cells were fixed with 95% ethanol at room temperature for 30 min. Cells were then pelleted and resuspended in 0.25% Triton X-100 in PBS on ice for 15 min. Following centrifugation, cells were resuspended in 10 μg/ml RNase A (Sigma) and 20 μg/ml propidium iodide (Biotium) in PBS for 30 min at room temperature in the dark. Cell cycle distribution was analyzed using a FACS Calibur flow cytometer and data analyzed using the FlowJo software (FlowJo LLC). All studies were repeated at least 3 times, data are reported as average ± S.D. and statistical significance was determined using an unpaired Student’s t test assuming unequal variance with p<0.05 considered significant.

### Cell extraction and ^1^H-MRS data acquisition

Approximately 1x10^7^ cells were extracted using the dual-phase extraction method as previously described [21]. Briefly, cells were trypsinized, centrifuged and vortexed in 10 ml of ice-cold methanol followed by 10 ml each of ice-cold chloroform and ice-cold water. Following phase separation, the aqueous phase was lyophilized and resuspended in 400 μL of deuterium oxide (Cambridge Isotope Laboratories, Andover, MA, USA)-based potassium phosphate buffer at pH 7. 5 mM sodium 3-(trimethylsilyl)propionate-2,2,3,3-d4 (TSP) (Cambridge Isotope Laboratories, Andover, MA, USA) placed in a coaxial insert was used as an external chemical shift and quantification reference.

Spectra were recorded using a 600 MHz Bruker Avance spectrometer (Bruker Biospin, Rheinstetten, Germany). Spectra were acquired using a 90° pulse, 3 second relaxation delay, 8 dummy scans, 16K data points, and 256 acquisitions (acquisition time ≈13 min). Water suppression was achieved using the 1D water presaturation ZGPR sequence. Prior to Fourier transformation, the FIDs were multiplied by an exponential weight function corresponding to a line broadening of 0.3 Hz.

### Spectral Processing and Statistical Analysis

The data is available at http://dx.doi.org/10.6084/m9.figshare.1226682

MRS data processing and statistical analysis were performed using the Metabonomic R package [22]. The chemical shift region from 4.60 to 4.90 ppm was excluded from the analysis to remove the random effects of variation in the residual water resonance suppression. Spectra were aligned to TSP signal, phase adjusted and normalized to the area of the TSP resonance and to the number of cells. Baseline correction was performed automatically using the Rocke and Xi method [23].

First, spectral data were centered and Pareto scaled [24] and Principal Component Analyses (PCAs) were then applied to the spectral data (N = 7 per IDH1 status in U87 cell lines and N = 6 per IDH1 status in NHA cell lines) to extract the most discriminative spectral subset from the total pool of metabolites. Hotteling’s T2 tests [25] were then applied over PCA loadings to select the resonances that significantly (p<0.05) discriminated between IDHwt and IDHmut samples. An unpaired Student’s t test was also applied to the full spectral data as a complementary analytical approach. Resonances determined as significantly different between IDHwt and IDHmut cells were identified using the Human Metabolome Database [26] and Chenomx NMR Suit (rel. 7.7) database.

In addition, the resonances identified as significantly different by PCA loadings analysis were individually integrated for metabolic quantification using the Global Spectral Deconvolution algorithm of MestRenova v. 8.1 (Mestrelab Research S.L., Santiago de Compostela, Spain). For the metabolic quantification (also N = 7 per IDH1 status in U87 cell lines and N = 6 per IDH1 status in NHA cell lines) data are reported as average ± S.D. and statistical significance was determined using an unpaired Student’s t test assuming unequal variance with p<0.05 considered significant.

## Results

**Fig 1:**
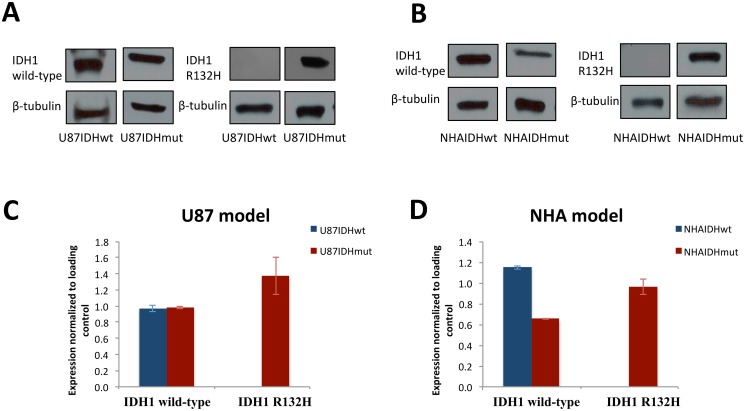
Western blots indicate that IDH1 protein expression is comparable between IDHwt and IDHmut cells. U87IDHwt and U87IDHmut cells (A) and NHAIDHwt and NHAIDHmut cells (B) were analyzed for expression of the IDH1 wild-type and R132H mutant proteins. β-tubulin was used as the loading control. Quantification of the results from 3 independent experiments for U87 (C) and NHA (D) models are shown.

First, we used Western blot analysis to assess the expression of IDH1 wild-type and R132H mutant proteins in our models (Fig. 1). As expected, there was no expression of the mutant enzyme in the IDHwt cell lines. In addition, we confirmed that the IDHwt and IDHmut cells display comparable protein expression i.e., the expression of the IDH1 wild-type protein in U87IDHwt and NHAIDHwt cells was comparable to the expression of the IDH1 R132H mutant protein in their IDHmut counterparts (representative blots are shown in Fig. 1 A and 1 B and quantification of the expression levels for the two cell lines is shown in Fig. 1 C and Fig. 1 D). We also confirmed that there was considerable expression of the IDH1 wild-type protein in the mutant cell lines, and that this expression was comparable to that of the IDH1 R132H protein.

Next, we examined the cell cycle profiles and the doubling times of the cell lines used in order to rule out an association between our metabolic findings and cell cycle distribution or doubling time. We found that in the U87 model, presence of mutant IDH1 led to a slight drop in G1 content (from 77.8 ± 0.2% to 67.3 ± 0.3) with a concomitant increase in G2 plus S content. In contrast, in the NHA model there was a slight increase in G1 content (from 46.6 ± 2.1% to 60.8 ± 0.7) with a concomitant drop in G2 plus S content. No apoptosis (as a sub-G1 peak in the cell cycle analysis and/or as floating cells in the tissue culture flask) could be detected in either model system. The doubling time of the U87IDHmut cells was unchanged at 27.2 ± 1.1 hours compared to 28.9 ± 0.5 hours for the U87IDHwt cells (p>0.05). Similarly, the doubling time of the NHAIDHmut cells, which was 37.7± 4.6 hours, was comparable to that of the NHAIDHwt cells, which was 40.2± 1.5 hours (p>0.05).

**Fig 2:**
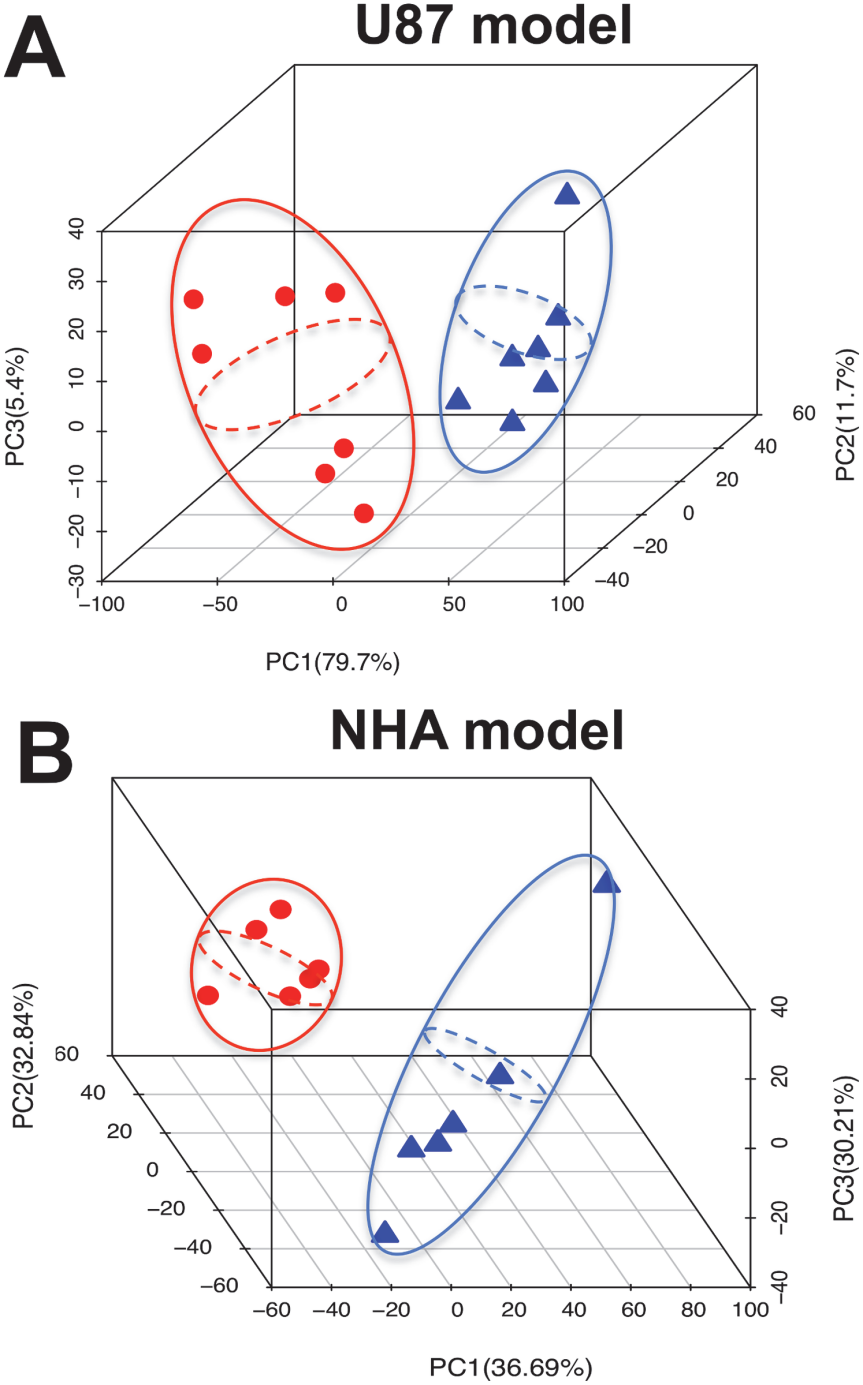
Score Plots of PCA reveal that IDHwt and IDHmut groups are clearly separated. The score plots of the PCA applied to the ^1^H-MRS spectra of cell extracts from IDHwt (Δ blue) and IDHmut (Ο red) cells for U87 (A) and NHA (B) models.

**Fig 3:**
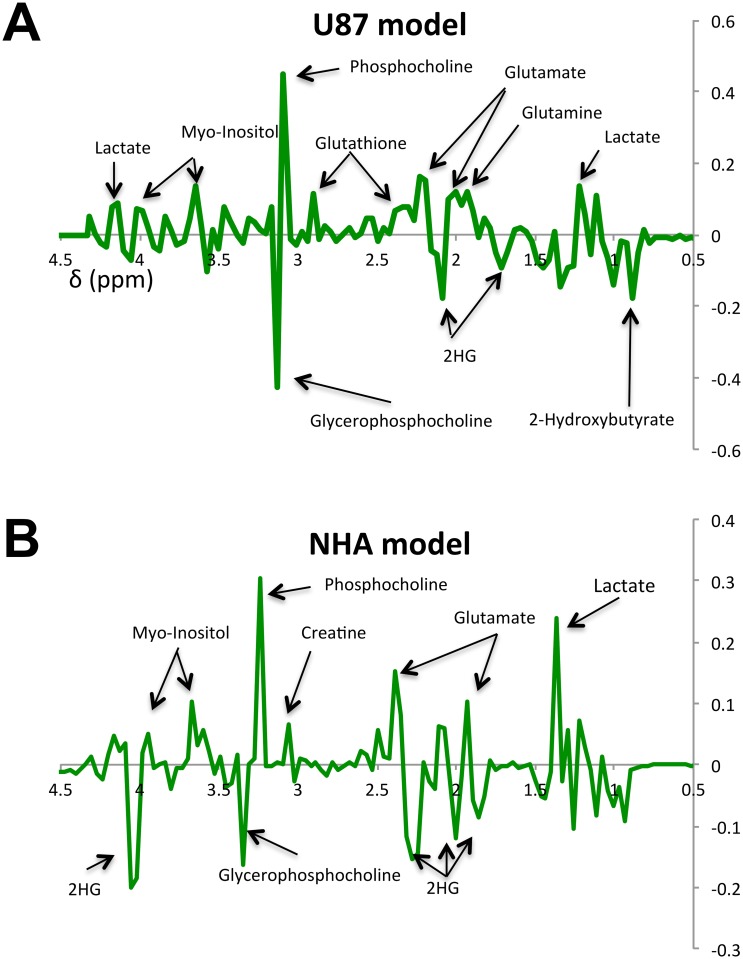
PCA loading plots identify metabolites that discriminate between IDHwt and IDHmut cells. Loading plots of PCA performed on the ^1^H-MRS spectra are displayed for U87 (A) and NHA (B) cell extracts. The most significant resonance for separation between IDHwt and IDHmut cells are selected based on a significance level lower than 5.00e-02 by Hotteling’s T2 test.

**Fig 4:**
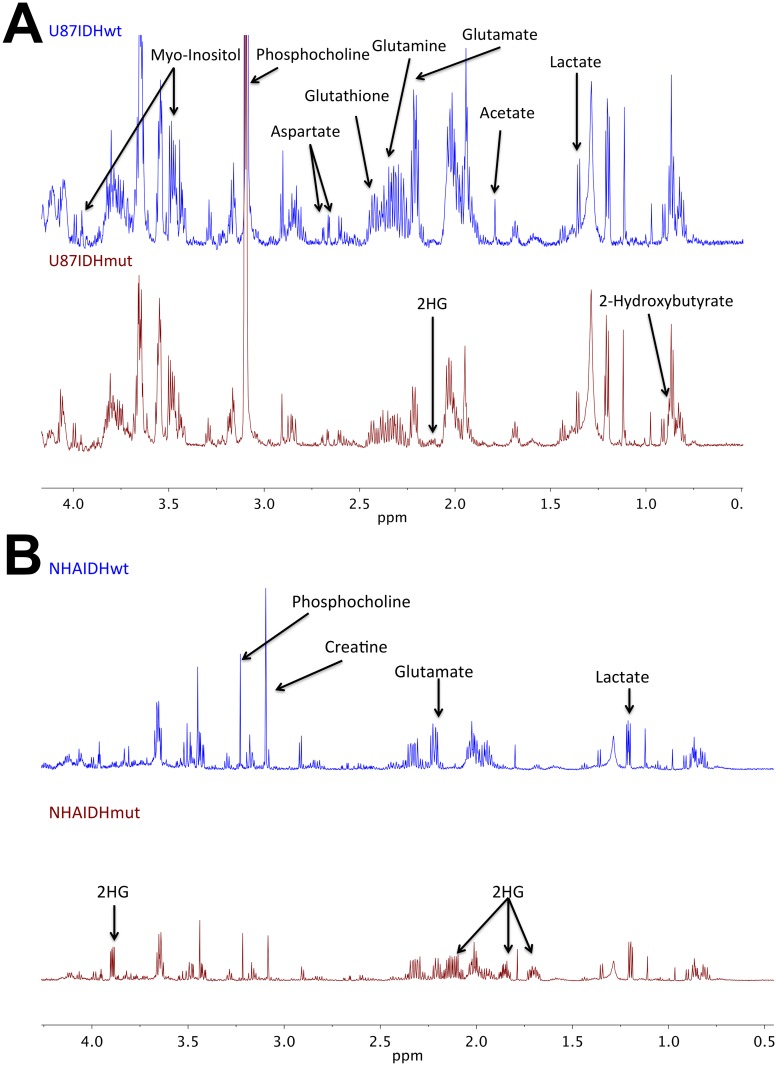
Metabolic differences between IDHwt and IDHmut cells are highlighted by the PCA. The representative aliphatic portions of the ^1^H-MRS spectra of IDHwt (blue, top panel) and IDHmut (red, bottom panel) cell extracts are shown for U87 (A) and NHA (B).

**Fig 5:**
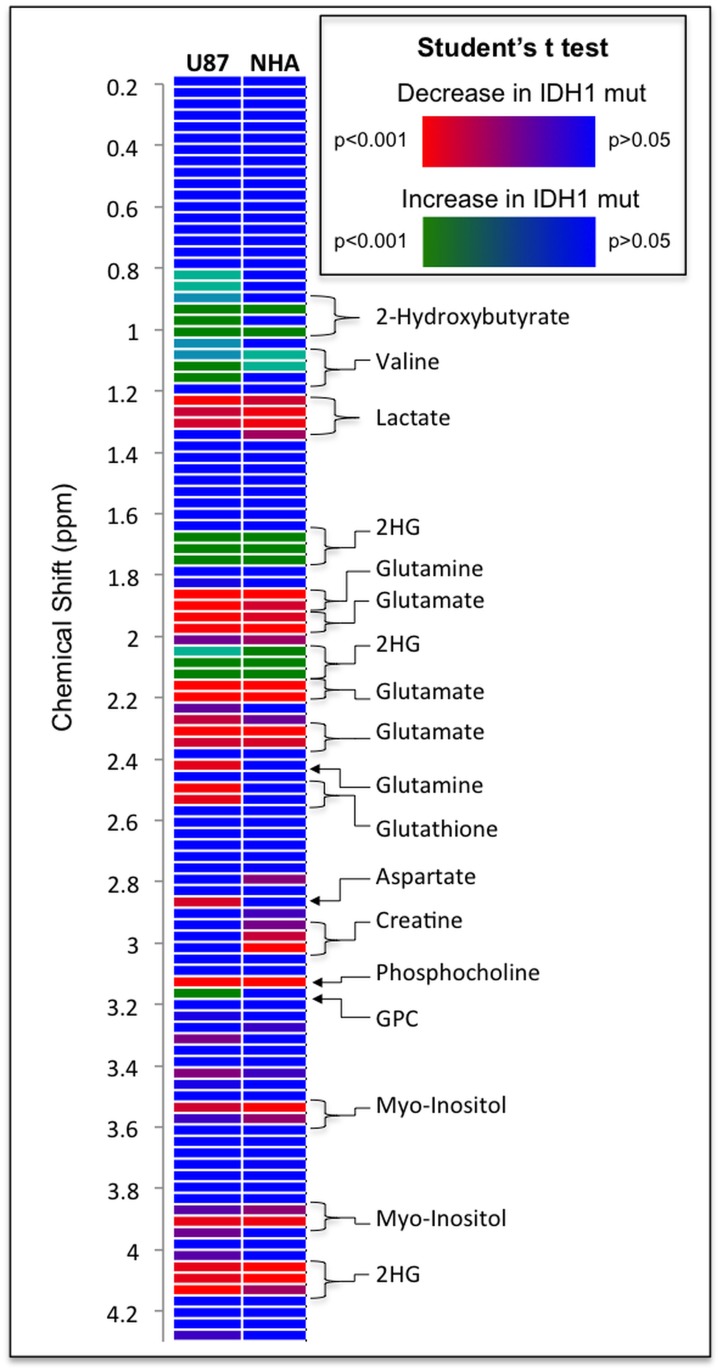
Chemical shift positions and the modulation of peak intensities resonating at that chemical shift when comparing IDHwt and IDHmut U87 and NHA cells. The color scale reflects the results of an unpaired Student’s t test applied to the spectral data. All non-significant changes (P > 0.05) are shown in blue, the significant increases and significant decreases in IDHmut cells are shown in green and red respectively.

We then extracted metabolites from the different cell lines, probed the cell extracts by MRS (see http://dx.doi.org/10.6084/m9.figshare.1226682 for the full data sets) and used unsupervised classification studies with PCA to analyze, in an unbiased way, the metabolic differences between IDHwt and IDHmut cells. As illustrated in Fig. 2, the scores plots obtained from the analysis provided clear discrimination based on the first principal component between IDHwt and IDHmut cells for both U87 and NHA models. Analysis of the loadings plots obtained along the first principal component (Fig. 3) from each of the PCAs was then used to identify the specific metabolites that significantly discriminated between IDHwt and IDHmut cells. The metabolic differences elicited by the PCA are further highlighted in representative ^1^H-MRS spectra (Fig. 4). Some of the metabolic alterations were specific to only one model. A significant decrease in glutamine, glutathione and myo-inositol was observed in U87IDHmut cells when compared to U87IDHwt cells, but was not observed in the NHA model. Lower creatine concentration was observed in NHAIDHmut cells when compared to NHAIDHwt, but not in the U87 model. Importantly however, the loadings plots obtained from the comparison of IDHwt and IDHmut cells indicated that several metabolic alterations were common to both cell models. Specifically, IDHmut cells showed a lower concentration of glutamate, lactate and phosphocholine (PC) as well as higher concentration of glycerophosphocholine (GPC) and 2-HG in both U87 and NHA cells. In addition, we also applied an unpaired Student’s t test to the full spectral data and found that the results were similar to the PCA analysis (Fig. 5). Specifically, we found that both IDH1 mutant cell models present significantly higher NMR intensities at the following chemical shift positions: 0.96–1.04ppm (assigned to 2-hydroxybutyrate), 1.11–1.19ppm (assigned to valine) and 1.71–1.78ppm, 2.08–2.16ppm and 4.10–4.17ppm (all assigned to 2-HG). They also show a significant decrease at the following chemical shift positions: 1.26–1.34ppm (assigned to lactate), 1.90–2.01ppm (assigned to the combined glutamine and glutamate signals), 2.19–2.23 and 2.31–2.38 (assigned to glutamate) and 3.16ppm (assigned to PC). In addition, U87IDHmut cells show a significant increase in the NMR intensities at 3.20ppm (assigned to GPC) and a significant decrease at 2.46ppm (assigned to glutamine), 2.53–2.57ppm and 2.90ppm (both assigned to glutathione), and 3.58ppm and 3.95ppm (both assigned to myo-inositol). NHAIDHmut cells show a significant decrease in the NMR intensity at 3.02–3.05ppm (assigned to creatine). Any additional modulations observed using this approach, including a drop at 2.83ppm, 3.35ppm and 3.46ppm were not associated with any known metabolite and, as such, provided no additional information beyond the well-established and robust PCA analysis.

**Fig 6:**
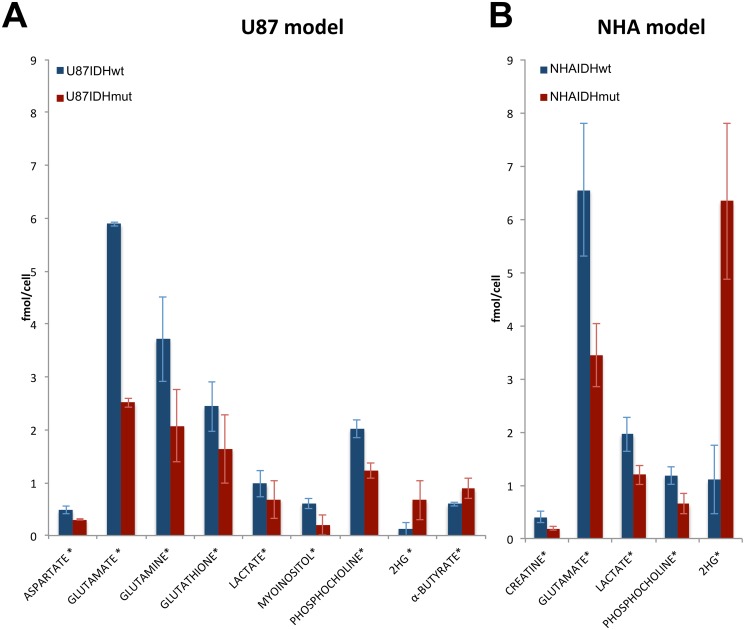
^1^H-MRS reveals the metabolic reprogramming associated with the IDH1 mutation. The intracellular concentration of the metabolites that differ significantly between IDHwt and IDHmut cells were quantified for U87 (A) and NHA (B) cell lines.

The absolute cellular concentrations of the metabolites identified as different on the PCA loading plots were then determined and, as illustrated in Fig. 6, the significance of changes in concentrations was further confirmed. As expected, 2-HG was present at a concentration of 0.67 ± 0.19 fmol/cell and 6.34 ± 1.47 fmol/cell in the U87IDHmut and NHAIDHmut cells respectively (it is not possible to distinguish between the IDH1 mutant-derived D-2-HG and the IDH1-independent L-2-HG stereoisomers, and thus our measurements of 2-HG reflect the total of both stereoisomers). 2-HG was below detection level in the IDHwt cells. PC levels dropped by 39% from 2.02 ± 0.12 fmol/cell in U87IDHwt to 1.22 ± 0.37 fmol/cell in U87IDHmut cells (p<0.01) and by 40% from 1.18 ± 0.19 fmol/cell in NHAIDHwt to 0.65 ± 0.16 fmol/cell in NHAIDHmut cells (p<0.05). The concentration of glutamate decreased by 57% from 5.89 ± 0.80 fmol/cell in U87IDHwt to 2.51 ± 0.68 fmol/cell in U87IDHmut (p<0.01) and by 42% from 6.55 ± 0.60 fmol/cell in NHAIDHwt to 3.45 ± 1.24 fmol/cell in NHAIDHmut cells (p<0.05). Finally lactate levels dropped by 30% from 0.98 ± 0.10 fmol/cell in U87IDHwt to 0.68 ± 0.19 fmol/cell (p<0.05) in U87IDHmut cells and by 28% from 1.96 ± 0.18 fmol/cell in NHAIDHwt to 1.20 ± 0.32 fmol/cell in NHAIDHmut cells (p<0.01). The increase in GPC that was observed for both models based on the PCA loading was not statistically significant when absolute cellular concentrations were measured.

As an added control, we also studied the metabolome of parental U87 and NHA cells. Importantly, no significant differences were found in the cellular concentrations of the metabolites analyzed in this study when comparing the parental cell lines with the IDHwt cell lines for both U87 and NHA models, underscoring the fact that the metabolic changes reported here result from the IDH1 mutation.

## Discussion

In this study we have characterized the metabolic profile of two genetically engineered IDH1 mutant cell models using ^1^H-MRS and a completely untargeted multivariate statistical analysis.

We chose to perform this study on genetically engineered cells because they provided a platform to compare stably transfected cells that differed only in the expression of mutant IDH1, and thus could serve to identify MR-detectable metabolic changes specifically associated with the IDH1 mutation. We confirmed that expression of mutant IDH1 did not alter doubling time or cell cycle distribution in a common manner in both of our cell models, and thus ruled out those factors as explanations of our findings. We also confirmed that in both of our model systems IDHmut cells express both wild-type and mutant IDH1 enzymes, and that the expression of these two proteins was comparable. Thus our models are consistent with clinical data that shows heterozygous expression of the mutant IDH1 gene, and are consistent with previous studies [10,11,20]. Furthermore, there should be no bias in our metabolomic analyses resulting from differentially skewed protein expression in the different cell lines.

It should be noted that previous studies by Turcan et al. [10] and Lu et al. [27] utilized normal human astrocytes of the same origin as in our current study. Lu et al. [27] report that trimethylation of histone H3 at lysine 9 was significantly elevated by passage 12 following transfection with the mutant IDH1 gene. Turcan et al. [10] compared the average methylation level of NHAIDHwt and NHAIDHmut cells and showed that while the methylation level remains unchanged over time for the NHAIDHwt cells, the average methylation level increases for the NHAIDHmut cells over time reaching greater than half maximal levels by passage 15. All our experiments were conducted at a passage level greater than 15. Therefore, we believe that the IDHmut cells we have used in our studies have had sufficient time to develop a hypermethylated phenotype, and thus our findings are associated with presence of the IDH1 mutation. Of note, previous work did note probe the cellular metabolism of these cells.

Metabolomic studies are typically performed using either MRS (also called nuclear magnetic resonance or NMR) or mass spectrometry (MS) [28]. MS is a highly sensitive method that can provide metabolomic information on hundreds of metabolites with pico- to femtomolar sensitivity. However, absolute quantification and identification of compounds can be challenging. More importantly, MS is destructive and, as such, has limited translational potential. In contrast, the sensitivity of ^1^H-MRS is lower, typically in the millimolar to micromolar range, and thus MRS detects less than a hundred metabolites [29]. For example, in our study we detected 41 metabolites, but did not have the sensitivity to probe for some of the 2-HG-associated metabolites such as isocitrate or α-KG. Nonetheless, MRS is a useful method to analyze metabolomics data by untargeted multivariate statistical methods [17,18] because of its highly quantitative and reproducible properties and because 2-dimensional MRS sequences are excellent tools for full metabolic identification based on the connectivity between MRS signals [30]. Most importantly, MRS is a nonradioactive and nondestructive method and can therefore be used noninvasively in the clinic. Indeed, methods for noninvasive *in vivo* monitoring of 2-HG in patients have been developed [31–33]. Probing for additional metabolic alterations that are associated with the IDH1 mutation can enhance such clinical investigations. Our studies, which provide high-resolution spectra of a homogenous population of IDHwt and IDHmut cells, can serve to identify such translational MR-detectable IDH1 mutation-specific metabolic alterations.

The overall profile of our two cell models, as illustrated by their spectra, was somewhat different likely reflecting their different genetic backgrounds: a glioblastoma cell line (U87) and an immortalized astrocyte cell line (NHA). The different genetic backgrounds could also explain why not all the metabolic alterations associated with the expression of the mutant IDH1 gene were identical in our two models. Nonetheless, three common metabolic changes were observed, in addition to the expected increase in total 2-HG. Glutamate, lactate, and PC, all dropped significantly in the IDHmut cells compared to IDHwt, most likely reflecting metabolic reprogramming that results from the expression of the mutant IDH1 enzyme. These findings are consistent with the findings reported by Reitman *et al*. [11] who investigated a genetically engineered human oligodendroglioma cell model using MS and a metabolomics approach. They found a significant drop in glutamate, glutathione, aspartate and PC, and an increase in 2-HG and GPC. The IDH1 associated metabolic changes described in this study have, therefore, been observed in three different genetically engineered cell models, using two different analytical methods in two independent studies. This serves to confirm the generality of the metabolic reprogramming that occurs in IDHmut cells.

Our findings are not entirely consistent with a previous report by Elkhaled *et al*. [12] in which an HR-MAS analysis of *ex vivo* low-grade glioma tumors showed a correlation between 2-HG and several metabolite levels including lactate and PC. One possible explanation for this discrepancy is that our studies specifically compare IDHwt to IDHmut cells, whereas the HR-MAS study was investigating only IDHmut samples. Some of the biopsy findings could therefore reflect differences in tumor cellularity within the tissue sample. Alternatively, because the IDH1 mutation is known to be an early event in tumor development, it is possible that the biopsy findings reflect additional events that occur during tumor development subsequent to the IDH1 mutation and that are not reflected in our models.

The decrease in glutamate concentrations observed in our cell models is consistent with the documented production of 2-HG from glutamine-derived α-KG through glutamate [7]. In the U87 model other metabolic alterations associated with this pathway were also observed, including a lower concentration of glutamine, which is in equilibrium with glutamate, and a lower concentration of glutathione, which is also derived from glutamine.

Glutamate is in equilibrium with α-KG and several enzymes that control that equilibrium, including the branched chain aminotransferases (BCAT1 and BCAT2), glutamate dehydrogenases, aspartate transaminases (encoded by the GOT1, GOT2 and GOT1L genes), and alanine transaminases (encoded by the GPT and GPT2 genes) influence the cellular concentration of glutamate. In the case of our U87 model, we recently showed that the cellular enzyme activity of BCAT, glutamate dehydrogenase, and aspartate transaminase, as well as the expression of BCAT1 and aspartate transaminase 1, were reduced in the IDHmut cells when compared to the IDHwt cells [34]. These findings are also consistent with an earlier study demonstrating hypermethylation of BCAT1 in U87IDHmut cells with a concomitant reduction in glutamate secretion into the media [35]. In the case of the NHA model, we have not investigated the expression or activity of these enzymes, but we mined the methylome data previously published by Turcan et al. [10] and which investigated immortalized astrocytes of the same origin as our NHAs. We looked at the dataset of 3141 genes that were found to be hypermethylated in mutant IDH1-expressing NHAs, and discovered that BCAT1, BCAT2, GOT1L1, GPT, GPT2 are all hypermethylated in the NHAIDHmut cells (5.8, 23.1, 14.8, 8.4 and 11.8 fold change in IDHmut vs. IDHwt respectively, see Table 1). Taken together, these findings suggest that hypermethylation and repression of several genes likely contributes to the observed decrease in glutamate production in IDHmut cells.

**Table 1:** The list of differentially methylated genes in mutant IDH1 expressing NHAs was downloaded from the supplementary information provided in the Turcan et al. paper [9] and mined for genes associated with the metabolites identified to be altered in mutant IDH1 cells in our study.

Probeset ID	Symbol	Normalized fold change (IDHmut/ IDHwt)	Delta-beta (P40 mut vs P40 wt)	Relation to UCSC CpG island	Q value
**cg16490209**	**BCAT1**	**5.8**	**0.1885695**	**N_Shelf**	**0.0463618**
**cg11956052**	**BCAT2**	**23.1**	**0.2472525**	**N_Shore**	**0.0426788**
**cg01505421**	**CHKB**	**1.5**	**0.01728**	**N_Shore**	**0.0437283**
**cg18555289**	**GPT**	**8.4**	**0.132311**	**N_Shore**	**0.0382745**
**cg00270789**	**GPT2**	**11.8**	**0.251366**	**S_Shore**	**0.0382745**
**cg00242020**	**GOT1L1**	**14.8**	**0.1955245**		**0.0417852**
**cg03177631**	**LDHA**	**1.3**	**0.00801755**	**Island**	**0.0391234**

The decrease in lactate concentration in IDHmut cells is consistent with our previous study, which investigated the status of lactate dehydrogenase A (LDHA, the enzyme that catalyzes the production of lactate from pyruvate) in mutant IDH1 patient samples and in patient-derived cell lines [36], and which demonstrated silencing of the LDHA gene by methylation. Hypermethylation and repression of the LDHA gene was also observed in the Turcan study [10] in the NHAIDHmut cells (1.3 fold IDHmut/ IDHwt, Table 1). However, it should also be noted that the data presented here reflects intracellular lactate levels only. Lactate is normally exported into the extracellular environment and this study did not investigate extracellular lactate levels.

PC and GPC have well-documented roles in membrane phospholipid synthesis [37] and breakdown [38]. In the context of cancer, elevated PC has been correlated with transformation and increased tumor aggressiveness [39]. PC and GPC levels also appear promising as predictors of glioma grade [40], with elevated PC levels being characteristic of high grade gliomas and GBMs, and elevated GPC levels being more characteristic of low grade gliomas. Thus the decrease in PC in our cell models and the trend towards an increase in GPC (detected by PCA loadings) are consistent with the metabolic profile of a low-grade tumor.

Choline phosphorylation to generate PC is catalyzed by choline kinase alpha and/or beta. Choline kinase alpha is typically overexpressed in cancer and is viewed as playing a central role in mediating the increase in PC levels that is observed in most tumors when compared to normal tissue [39]. Interestingly, when considering the Turcan et al. study [10], the methylome data indicates that CHKB, the gene coding for choline kinase beta, is hypermethylated in mutant IDH1 cells (1.5 fold IDHmut/IDHwt, Table 1) and thus its associated transcriptional repression could account for the reduction in PC levels in IDHmut cells. Nonetheless, multiple other anabolic and catabolic enzymes could be involved in modulating PC as well as GPC levels and, importantly, not only expression, but also localization, post-translational modifications and co-factor levels can affect cellular enzyme activities. Thus, further studies are needed to fully assess the mechanism through which presence of the mutant IDH1 gene changes the levels of choline containing metabolites.

In summary, by investigating the snapshot of steady state cellular metabolite levels, our work identifies three different metabolites that, in addition to 2-HG, are consistently modulated by the IDH1 mutation in different cell models. As such, these findings could serve to validate and enhance clinical *in vivo* findings, highlighting the metabolic changes specifically associated with the IDH1 mutation. Our study also paves the way for future research directions that could lead to a greater understanding of the metabolic reprogramming associated with the IDH1 mutation, allowing the development of new therapeutic approaches and associated metabolic imaging biomarkers.
